# Event and Comment

**Published:** 1917-07

**Authors:** 


### DENTAL REGISTER

Vol. LXXI JULY, 1917 No. 7

### EVENT AND COMMENT

PATRIOTIC INFLAMMATION. This is a time of much unrest on account of the extraordinary issues which are to be met and properly adjusted. The dental profession like all other callings will need to do some careful thinking and act most soberly to avoid making serious blunders consequent on acting hastily, or under the excitement of the stirring events of preparations for taking up our part in this great world war. It is not yet quite clear just what part the dental profession will be called upon to assume the responsibility of maintaining. Our work should be closely attached to that of the medical profession, and we should be ready to take our place when it is clearly assigned to us. In the first place, it should not be expected that we can rush into war service without adequate leadership. We know from information that comes to us that England and France made the mistake of allowing their physicians and dentists to enlist in large numbers in the regular military service; and now, because of accidents in the battle front, they are woefully lacking in medical service, both for the army and civilian populations in those countries. This happened because the medical and dental professions in those countries were carried away with so much loyal patriotism that they did not take time to look ahead to the time that has actually overtaken them, when it is necessary to have a reserve corps that can repair the damages done to their soldiers so that they can be returned to duty at the front. They are now making heavy demands on the medical resources of this country, and unless we face this issue courageously and wisely we shall be short of similar resources before we know it. Canadian dentists have managed this matter better and we shall do well to follow their example so far as possible. They perfected an organization and selected competent leaders who have had the support of the Canadian Government, and it is safe to say, judging from the reports at hand, that their work abroad is most efficiently done, and they are in good condition to carry out their program to the end of the war. It seems to us that what we need more than anything else now is just such competent leadership. There should be established as promptly as possible a competent dental leadership that has a definite authority, that will put the dental corps into the army on a definite basis. At present we hear of all sorts of dentists getting prepared for service and willing to leave important work at home to serve in the army. Some are fit and others not. Students just graduating from college can do some kinds of dental service, but they can not, for lack of experience, be delegated to important surgical or prosthetic restorative service. Some of the men offering are not physically qualified, and others are needed at home more than in the army. The teachers in our schools should not be taken away, as this war may last many years and we shall need more dentists in the next few years than ever before. Therefore it appears to be a time for thoughtful preparation rather than for reckless or extravagant squandering of our resources. We all want to do our part, but for the most part we don't know what our part should be, and there is danger that out of our enthusiasm we may do the wrong thing. We have already a committee which is cooperating with the surgeon general of the army, and if we can only be patient enough to wait until this committee can say definitely what it is that we can do to the best advantage it will be better than for the individual or even the so-called units to act independently. There is so much anxiety and impatience manifested that we hope this committee may gets its plans before the profession at as early a date as possible. In the meantime let us refrain from acting precipitately, and without some definite proposition that might prevent a thorough and efficient organization that will bring credit tc our profession because of efficient service. In short, let us resolve that we are in this war to see it through, and that it will be infinitely wiser for us to cooperate on a definite plan than to spend ourselves quickly in personal endeavors. Efficiency in this business should result most surely from cooperation in an organized effort. Our excitement is destructive in its tendencies, and we need to be determinedly constructive.

